# Observed Incidence of Uveitis Following Certolizumab Pegol Treatment in Patients With Axial Spondyloarthritis

**DOI:** 10.1002/acr.22848

**Published:** 2016-05-26

**Authors:** M. Rudwaleit, J. T. Rosenbaum, R. Landewé, H. Marzo‐Ortega, J. Sieper, D. van der Heijde, O. Davies, H. Bartz, B. Hoepken, T. Nurminen, A. Deodhar

**Affiliations:** ^1^ Klinikum BielefeldBielefeldGermany; ^2^ Devers Eye Institute, Legacy Health System, Portland, Oregon, and Oregon Health & Science UniversityPortland; ^3^ Academic Medical Center, Amsterdam and Atrium Medical CenterHeerlenthe Netherlands; ^4^ Leeds Teaching Hospitals NHS Trust and Leeds Institute of Rheumatic and Musculoskeletal Medicine, University of LeedsLeedsUK; ^5^ University Hospital CharitéBerlinGermany; ^6^ Leiden University Medical CentreLeidenthe Netherlands; ^7^ UCB PharmaSloughUK; ^8^ UCB PharmaMonheimGermany; ^9^ Oregon Health & Science UniversityPortland

## Abstract

**Objective:**

Axial spondyloarthritis (axial SpA) is characterized by inflammation of the spine and sacroiliac joints and can also affect extraarticular sites, with the most common manifestation being uveitis. Here we report the incidence of uveitis flares in axial SpA patients from the RAPID‐axSpA trial, including ankylosing spondylitis (AS) and nonradiographic (nr) axial SpA.

**Methods:**

The RAPID‐axSpA (NCT01087762) trial is double‐blind and placebo‐controlled to week 24, dose‐blind to week 48, and open‐label to week 204. Patients were randomized to certolizumab pegol (CZP) or placebo. Placebo patients entering the dose‐blind phase were re‐randomized to CZP. Uveitis events were recorded on extraarticular manifestation or adverse event forms. Events were analyzed in patients with/without history of uveitis, and rates reported per 100 patient‐years.

**Results:**

At baseline, 38 of 218 CZP‐randomized patients (17.4%) and 31 of 107 placebo‐randomized patients (29.0%) had past uveitis history. During the 24‐week double‐blind phase, the rate of uveitis flares was lower in CZP (3.0 [95% confidence interval (95% CI) 0.6–8.8] per 100 patient‐years) than in placebo (10.3 [95% CI 2.8–26.3] per 100 patient‐years). All cases observed during the 24‐week double‐blind phase were in patients with a history of uveitis; in these patients, rates were similarly lower for CZP (17.1 [95% CI 3.5–50.1] per 100 patient‐years) than placebo (38.5 [95% CI 10.5–98.5] per 100 patient‐years). Rates of uveitis flares remained low up to week 96 (4.9 [95% CI 3.2–7.4] per 100 patient‐years) and were similar between AS (4.4 [95% CI 2.3–7.7] per 100 patient‐years) and nr‐axial SpA (5.6 [95% CI 2.9–9.8] per 100 patient‐years).

**Conclusion:**

The rate of uveitis flares was lower for axial SpA patients treated with CZP than placebo during the randomized controlled phase. Incidence of uveitis flares remained low to week 96 and was comparable to rates reported for AS patients receiving other anti–tumor necrosis factor antibodies.

## INTRODUCTION

Uveitis is the most common extraarticular manifestation experienced by patients with axial spondyloarthritis (SpA) 1, and affects patients with both ankylosing spondylitis (AS) and nonradiographic axial SpA (nr‐axial SpA) 2. While limited information is available regarding the overall axial SpA population, 1 study reported the prevalence of ever experiencing a uveitis flare as 19.3% for AS patients with disease duration of ≤5 years and as 12.4% for nr‐axial SpA patients 3. Reports from patients with established AS suggest that ∼40% of patients will experience 1 or more uveitis flares during the course of their disease 4, and observational data sources (such as cohort studies and patient registries) have reported that 10–30% of axial SpA patients classified using the Assessment of SpondyloArthritis international Society (ASAS) criteria have a history of uveitis 2, 5. Moreover, an attack of acute anterior uveitis (AU) can be the first presenting symptom that leads to a diagnosis of axial SpA 6.

Box 1 **Significance & Innovations**

- The incidence of uveitis flares during the 24‐week, double‐blind, placebo‐controlled period of the RAPID-axSpA trial was lower for axial SpA patients treated with certolizumab pegol (CZP) than with placebo.
- In patients with a prior history of uveitis, the uveitis flare rate was also lower for axial SpA patients treated with CZP than placebo during the double‐blind period.
- The reported uveitis event rate remained low with increased exposure to CZP throughout both the dose‐blind and open‐label study periods to week 96.
- The incidence of uveitis flares observed with CZP treatment of axial SpA patients, including both ankylosing spondylitis (AS) and nonradiographic axial SpA patients, was comparable to the incidence rates reported for AS patients treated with other anti–tumor necrosis factor antibodies.

The burden of uveitis on affected patients is high, as it is commonly associated with photophobia, pain, and in some cases blurred vision 6. In axial SpA patients, AU can range from a single flare to an episodic and recurrent course. Flares of uveitis are typically of sudden onset in a unilateral fashion 7. Regardless of disease course, in patients developing uveitis, impaired visual performance and reduced health‐related quality of life is reported 8. Acute attacks usually resolve completely with topical corticosteroid treatment. For axial SpA patients who develop episodic and recurrent uveitis flares, treatment is limited. Nonsteroidal antiinflammatory drugs (NSAIDs) may relieve symptoms for a short period of time 9, and the disease‐modifying antirheumatic drugs sulfasalazine and methotrexate have been used, although available evidence relating to a possible decrease in flares of uveitis is limited to a low number of observational reports and small clinical trials 10, 11, 12, 13, 14. Given the lack of treatment options and the fact that a substantial proportion of such patients are resistant to conventional therapies, new treatment avenues have been explored to gain control of uveitis flares in these patients 15.

Analyses of uveitis flares from multiple randomized controlled trials (RCTs), in which patients received anti–tumor necrosis factor (TNF) therapy for the treatment of AS, have demonstrated a significant reduction in the incidence of acute AU flares in AS patients receiving etanercept 16 or infliximab compared to placebo 17. Furthermore, data from a large, open‐label noncontrolled study demonstrated a significant reduction in the rate of AU flares following adalimumab treatment of AS patients 18. A pooled data analysis 17 and recent review 19 have suggested that the monoclonal antibodies, adalimumab and infliximab, are equally effective in reducing AU flares and are superior to etanercept in this regard. However, at present, there are no RCT data available demonstrating the efficacy of anti‐TNF therapy in the reduction of uveitis flares in an axial SpA population (i.e., containing both AS and nr‐axial SpA patients).

Certolizumab pegol (CZP), a PEGylated Fc‐free anti‐TNF, has been shown to improve spinal and extraarticular manifestations, such as enthesitis, in patients with axial SpA (including both AS and nr‐axial SpA patients) 20. Emerging data from a retrospective case series posit a treatment effect of CZP in active refractory uveitis, which included 2 patients with AS from a series of 7 21. However, further data are required to determine the effectiveness of CZP in reducing the incidence of uveitis flares in axial SpA patients, an important facet of disease symptomatology 22.

Here we provide a comprehensive summary of the data relating to the incidence of uveitis flares in CZP‐treated patients with axial SpA from the RAPID‐axSpA trial; this is the first study to explore the effects of an anti‐TNF on comorbid uveitis in the broad population of patients with axial SpA, including AS and nr‐axial SpA patients.

## MATERIALS AND METHODS

#### Patients

Detailed inclusion and exclusion criteria for the RAPID‐axSpA trial have been reported previously 23. In brief, eligible patients were age ≥18 years and had active disease (Bath Ankylosing Spondylitis Disease Activity Index score and spinal pain score ≥4), an inadequate response to ≥1 NSAID, and fulfilled the ASAS criteria for adult‐onset axial SpA.

In the RAPID‐axSpA trial, patient randomization was stratified by fulfillment of the modified New York criteria and included patients with AS (radiographic sacroiliitis as defined by the modified New York criteria: 54.8%, 178 patients) and nr‐axial SpA, as defined by the ASAS criteria (no radiographic sacroiliitis: 45.2%, 147 patients).

#### Study design

The RAPID‐axSpA trial (NCT01087762) is a phase 3, multicenter trial in axial SpA patients and is double‐blind and placebo‐controlled to week 24, dose‐blind to week 48, and an open‐label (OL) to week 204.

Patients were randomized 1:1:1 to placebo, or CZP 400 mg at weeks 0, 2, and 4 (loading dose) followed by either CZP 200 mg every 2 weeks (Q2W) or CZP 400 mg every 4 weeks (Q4W). Patients originally randomized to CZP in the double‐blind phase continued on their assigned dose in the dose‐blind phase and OL. Placebo patients who were nonresponders according to ASAS20 response criteria at both weeks 14 and 16, or placebo patients who completed the 24‐week double‐blind phase, entered the dose‐blind phase and were re‐randomized 1:1 to CZP 200 mg Q2W or CZP 400 mg Q4W following the CZP loading dose.

#### Statistical analyses

Uveitis events were recorded on a specific case report form for extraarticular manifestations, or on adverse event forms using the Medical Dictionary for Regulatory Activities, version.14.1, preferred terms “uveitis,” “iritis,” or “iridocyclitis.” New incidences and flares were analyzed in patients with or without a history of uveitis, the former group being defined as patients with uveitis recorded on their standard medical history, ASAS classification criteria screening assessment, or baseline extraarticular assessment. Data are reported as uveitis event rates per 100 patient‐years, which are presented alongside exact Poisson fiducial confidence limits 24.

For week 24 analyses, the number of uveitis events was compared between those randomized to CZP (combined dose regimens) and placebo. For week 48 and week 96 analyses, all patients exposed to ≥1 dose of CZP were considered.

Patient‐level data were additionally collected where available and considered for each uveitis event. All types of uveitis events described herein are referred to as “flare.” Additional data reported include flare duration, severity, and treatment outcome. Flare severity was determined as mild, moderate, or severe based on individual investigator assessment.

Due to the post hoc nature of the analysis, including but not limited to the lack of pre‐specification of flare definitions, identification and stratification by uveitis history, and choice of potential statistical test methods, no statistical test was performed on the difference between the incidence of uveitis in the CZP and placebo groups.

## RESULTS

#### Patient disposition and baseline characteristics

At baseline, 218 patients were originally randomized to CZP and 107 to placebo. Of the 218 CZP‐randomized patients, 38 (17.4%) had a history of uveitis, as did 31 of the 107 placebo‐randomized patients (29.0%). The proportion of patients with a history of uveitis was similar in the AS and nr‐axial SpA subpopulations (20.8% and 21.1%, respectively). Baseline demographics and disease activity were similar between patients with and without a history of uveitis (Table 1). Although, a greater proportion of patients with a history of uveitis also had a history or current diagnosis of inflammatory bowel disease (10.1%) than those without a history of uveitis (4.3%) (Table 1).

**Table 1:** Baseline demographics and disease characteristics for all patients and for those with or without a history of uveitis^a^

	**All axial SpA patients (n = 325)**	**History of uveitis (n = 69)**	**No prior history of uveitis (n = 256)**
Age, mean ± SD years	39.6 ± 11.9	41.6 ± 11.5	39.1 ± 11.9
Male sex	200 (61.5)	41 (59.4)	159 (62.1)
CRP, median mg/ml	13.9	13.9	13.5
>Upper limit of normal, 7.9 mg/ml	235 (72.3)	52 (75.4)	183 (71.5)
BASDAI score, mean ± SD	6.45 ± 1.6	6.54 ± 1.5	6.42 ± 1.6
BASFI score, mean ± SD	5.38 ± 2.2	5.09 ± 1.9	5.46 ± 2.3
ASDAS, mean ± SD	3.84 ± 1.0	3.94 ± 0.8	3.87 ± 0.9
Peripheral arthritis^b^	124 (38.2)	25 (36.2)	99 (38.7)
Enthesitis^c^	229 (70.5)	48 (69.6)	181 (70.7)
Extraarticular features of axial SpA (patient history or current diagnosis), defined by ASAS classification criteria screening assessment			
Heel enthesitis	117 (36.0)	26 (37.7)	91 (35.5)
Psoriasis	20 (6.2)	5 (7.2)	15 (5.9)
Inflammatory bowel disease	18 (5.5)	7 (10.1)	11 (4.3)

a Values are the number (percentage) unless indicated otherwise. SpA = spondyloarthritis; CRP = C‐reactive protein; BASDAI = Bath Ankylosing Spondylitis Disease Activity Index; BASFI = Bath Ankylosing Spondylitis Functional Index; ASDAS = Ankylosing Spondylitis Disease Activity Score; ASAS = Assessment of SpondyloArthritis international Society.

b Defined as at least 1 swollen joint in a 44‐joint assessment.

c Defined as a Maastricht Ankylosing Spondylitis Enthesitis Score >0.

#### Incidence of uveitis

During the 24‐week placebo‐controlled period of the RAPID‐axSpA trial, there were no de novo cases of uveitis observed, i.e., all events were observed in patients with a history of uveitis. The rate of uveitis flares to week 24 was lower in patients treated with CZP (3.0 [95% confidence interval (95% CI) 0.6**–**8.8] per 100 patient‐years; 3 events in 3 patients) than with placebo (10.3 [95% CI 2.8**–**26.3] per 100 patient‐years; 4 events in 4 patients) (Table 2). This difference was maintained when considering only those patients with a prior history of uveitis (uveitis flare rate of 17.1 [95% CI 3.5**–**50.1] per 100 patient‐years for CZP‐treated patients, compared to 38.5 [95% CI 10.5**–**98.5] per 100 patient‐years for placebo) (Table 2). Long‐term incidence of uveitis data, collected to week 96 of the RAPID‐axSpA trial, demonstrated that over the dose‐blind (to week 48) and open‐label (to week 96) study periods the overall rate of uveitis flares remained similar to that observed over 24 weeks (4.9 [95% CI 2.5**–**8.6] per 100 patient‐years at week 48, and 4.9 [95% CI 3.2**–**7.4] per 100 patient‐years at week 96; 12 events in 11 patients, and 24 events in 23 patients, respectively) (Table 2). The rates observed in patients with a prior history of uveitis also remained stable to week 96 (20.8 [95% CI 10.0**–**38.3] per 100 patient‐years at week 48 [10 events in 9 patients] and 16.4 [95% CI 9.4**–**26.7] per 100 patient‐years at week 96 [16 events in 15 patients]) (Table 2). Additionally, there was little difference observed in uveitis flare rates between CZP dose regimens (data not shown).

**Table 2:** Incidence of uveitis flares in axial SpA patients treated with CZP or placebo to week 24, and with CZP to weeks 48 and 96^a^

	Uveitis flare rate (95% CI)^b^	Uveitis flares, no.	Patients with uveitis flares, no.	Exposure, patient‐years
Week 24				
CZP				
All patients (n = 218)	3.0 (0.6–8.8)	3	3	100.0
History of uveitis (n = 38)	17.1 (3.5–50.1)	3	3	17.5
No history of uveitis (n = 180)	0.0	0	0	82.5
Placebo				
All patients (n = 107)	10.3 (2.8–26.3)	4	4	38.9
History of uveitis (n = 31)	38.5 (10.5–98.5)	4	4	10.4
No history of uveitis (n = 76)	0.0	0	0	28.5
Week 48				
CZP^c^				
All axial SpA patients (n = 315)	4.9 (2.5–8.6)	12	11	244.1
History of uveitis (n = 63)	20.8 (10.0–38.3)	10	9	48.0
No history of uveitis (n = 252)	1.0 (0.1–3.7)	2	2	196.1
Week 96				
CZP^c^				
All axial SpA patients (n = 315)	4.9 (3.2–7.4)	24	23	485.7
History of uveitis (n = 63)	16.4 (9.4–26.7)	16	15	97.3
No history of uveitis (n = 252)	2.1 (0.9–4.1)	8	8	388.4

a SpA = spondyloarthritis; CZP = certolizumab pegol; 95% CI = 95% confidence interval.

b Per 100 patient‐years.

c Data shown are cumulative from week 0 for all patients independent of baseline randomization (treatment with either CZP dose regimen or placebo). Patients randomized to placebo at baseline received CZP from week 16 (if escaping early) or week 24 (if completing double‐blind phase).

Of 252 patients without prior history of uveitis, 8 experienced new‐onset uveitis flares from week 24 to week 96 of the study. Of the 24 events occurring to week 96, only 4 of these were coded as either “iritis” or “iridocyclitis,” with the remaining 20 events coded as “uveitis” or captured as such on patients’ extraarticular manifestation forms. The rates of uveitis flares at week 96 were also similar when considering patients’ modified New York classification at baseline. The uveitis flare rates were 4.4 (95% CI 2.3–7.7) per 100 patient‐years in the AS and 5.6 (95% CI 2.9–9.8) per 100 patient‐years in the nr‐axial SpA subpopulations (12 events in 12 patients for AS, and 12 events in 11 patients for nr‐axial SpA).

#### Uveitis flare duration, severity, and outcome

Additional patient‐level data were available and collected for 22 of the 24 uveitis events. These were reported from 21 patients, with 1 patient experiencing 2 events. Most of these events were reported as unilateral (13 of 22 events); the remaining 9 were unspecified. All reported events were either mild (9 of 22 events) or moderate (13 of 22 events) in severity, with none reported as severe and none leading to patient withdrawal from the trial. The median duration of uveitis flare was 27.5 days (interquartile range 9.0–55.0). One patient had a uveitis flare within 6 weeks of initiating CZP treatment. This patient had a prior history of uveitis and developed a mild episode of uveitis just 6 days after the first CZP injection. The patient recovered after 4 days and did not experience another episode of uveitis to week 96 of the study. The 8 patients with de novo cases of uveitis were ages 21–41 years, and the majority were male (6 of 8 patients) and HLA–B27 positive (5 of 8 patients). Five cases were unilateral in nature and 3 were unspecified. The new‐onset flares developed from 37 to 76 weeks after the 8 patients received their first CZP injection and from 4 days to 20 weeks after their latest CZP injection. All cases were mild to moderate in severity and all patients received concomitant medication for uveitis flare. One patient was not receiving CZP at time of flare onset (withdrawal); CZP dose was interrupted in 1 patient and CZP dose was unchanged in the remaining 6 patients. The patient whose CZP dose was interrupted was a 25‐year‐old white, HLA–B27‐positive male with nr‐axial SpA who had enthesitis and arthritis; the uveitis flare resolved. The patient reporting flare at the withdrawal visit recovered, and at week 96 only 1 patient had an ongoing flare. This patient was a 21‐year‐old African American, HLA–B27‐positive male with AS, who had a past medical history of dextroscoliosis, cardiomegaly, and dactylitis, and he remained in the study.

## DISCUSSION

In axial SpA, including both AS and nr‐axial SpA patients, uveitis is the most common extraarticular clinical manifestation 25 and contributes substantially to the overall burden of disease 26. Accordingly, the management of uveitis manifestations represents an important aspect of disease management 27.

Here we demonstrate that the proportion of patients with a prior history of uveitis, and the incidence of uveitis flares to week 96, was similar across the broad axial SpA population, including patients with AS and nr‐axial SpA. Patients treated with CZP had a uveitis event rate 71% lower than that observed for patients treated with placebo, although the number of events and affected patients was low and cannot be considered as statistically significant evidence of the inhibitory effect of CZP on uveitis flares. During the placebo‐controlled trial period, all uveitis flares occurred in patients with a past history of uveitis, and in these patients the difference between CZP and placebo uveitis event rates was retained. Long‐term data demonstrate that with continued CZP treatment the incidence of uveitis flares remained low and stable to week 96, with similar rates seen in both the AS and nr‐axial SpA subpopulations.

De novo cases of AU with anti‐TNF treatment have previously been reported 28 and it has been suggested that new‐onset cases are more frequent with etanercept than infliximab 29. In the RAPID‐axSpA trial, 8 patients experienced new‐onset flares of uveitis to week 96 of the study. The new‐onset cases of AU reported here share the same characteristics of a typical case of comorbid uveitis expected in a patient with axial SpA.

In this study, the incidence of uveitis flares in CZP‐treated axial SpA patients was comparable to rates observed for other anti‐TNF antibodies in AS patients. The AU flare rate of 4.9 per 100 patient‐years at weeks 48 and 96 was slightly higher than the pooled AU flare rate of 3.4 per 100 patient‐years reported in infliximab‐treated AS patients 17 and slightly lower than the AU flare rate of 7.4 per 100 patient‐years reported in adalimumab‐treated AS patients 18. By comparison, the reported AU flare rate in AS patients receiving treatment with etanercept was 12.0 per 100 patient‐years and 15.6 per 100 patient‐years for patients with AS receiving placebo in a pooled analysis 17. However, interpretation of these results is difficult given the different patient populations in each of the clinical trials, the low number of patients affected, and because definition of flare may differ between studies.

The data reported here add to our existing knowledge base supporting the use of anti‐TNF agents to prevent or reduce the incidence of axial SpA–associated uveitis flares 17, 18, 19, 30, 31.

A recent retrospective case series studied the efficacy and tolerability of CZP in refractory active uveitis with positive results 21. All 7 patients included in the series had failed treatment with at least 2 prior anti‐TNFs and, of the 7 patients, 2 had AS 21. While the number of patients was small, the data suggest that CZP may be effective in the treatment of refractory active uveitis 21, although further prospective data are needed to confirm these findings.

Baseline data from this study also support previous reports regarding the interrelationship between inflammatory disease of the spine/joints, skin, eye, and bowel. AS patients with inflammatory bowel disease were found to have a higher prevalence of iritis than controls 32, and inflammatory bowel disease has been shown to be independently associated with acute AU in AS patients 33. Here there was a higher rate of concurrent inflammatory bowel disease in patients with a history of uveitis than in those without.

The strengths of this study lie in the fact that data could be analyzed from a prospective and controlled trial with a 24‐week placebo period, which allowed the analysis of incidence rates of uveitis in axial SpA in an objective way. However, the analyses reported here have a number of limitations, including the low number of patients experiencing uveitis flares in the RAPID‐axSpA trial and the retrospective post hoc nature of the analyses. As a consequence, patients in the RAPID‐axSpA trial were not stratified by their prior history of uveitis, leading to an imbalance in the number of patients with prior history of uveitis between the CZP and placebo treatment groups.

The results of this study suggest that CZP has an impact on reducing uveitis flares in axial SpA patients, including both the AS and nr‐axial SpA subpopulations; however, further data are required from prospective studies to confirm these findings.

## AUTHOR CONTRIBUTIONS

All authors were involved in drafting the article or revising it critically for important intellectual content, and all authors approved the final version to be submitted for publication. Dr. Rudwaleit had full access to all of the data in the study and takes responsibility for the integrity of the data and the accuracy of the data analysis.

### Study conception and design

Sieper, Davies, Hoepken, Deodhar.

### Acquisition of data

Rudwaleit, Landewé, Marzo‐Ortega, van der Heijde, Davies, Hoepken, Deodhar.

### Analysis and interpretation of data

Rudwaleit, Rosenbaum, Landewé, Marzo‐Ortega, Sieper, van der Heijde, Davies, Bartz, Hoepken, Nurminen, Deodhar.

## ROLE OF THE STUDY SPONSOR

UCB Pharma sponsored the study and the development of the manuscript. In addition to content approval by the authors, UCB signed off on the manuscript following a full review to ensure that the publication did not contain any information that had the potential to damage the intellectual property of UCB. Joseph Burgon, PhD, and Sana Eljamel, MBChB, from Costello Medical Consulting, Cambridge, UK, provided medical writing and editorial assistance in preparing this manuscript for publication, based on the authors’ input and direction. All costs associated with the development of this manuscript were funded by UCB Pharma.
